# A Case of AIDS Diagnosed in the Intensive Care Unit with Concurrent Influenza Infection and *Pneumocystis jirovecii* Pneumonia

**DOI:** 10.1155/carm/5588716

**Published:** 2025-04-14

**Authors:** Yeliz Özdemir, İlhan Bahar, Gülfem Ece

**Affiliations:** ^1^Department of Infectious Diseases and Clinical Microbiology, Izmir City Hospital, Izmir, Türkiye; ^2^Department of Intensive Care Unit, TC Bakırçay University Cigli Training and Research Hospital, Izmir, Türkiye; ^3^Department of Medical Microbiology, Izmir City Hospital, Izmir, Türkiye

**Keywords:** HIV, influenza, opportunistic, *Pneumocystis jirovecii*

## Abstract

**Introduction:** People living with HIV (PLWH) are highly susceptible to respiratory infections, particularly pneumonia, which is often polymicrobial. A rapid decline in CD4 T lymphocytes, especially with concurrent influenza, increases the risk of *Pneumocystis jirovecii* pneumonia (PCP).

**Case Presentation:** This report discusses a newly diagnosed Acquired Immunodeficiency Syndrome (AIDS) patient with influenza and PCP coinfection, highlighting diagnosis, follow-up, and prognosis.

**Conclusions:** Community-acquired pneumonia is common in PLWH, especially among those not receiving antiretroviral therapies (ART). Co-infections with bacterial, viral, and fungal pathogens are common. Early identification of etiological agents and prompt treatment are crucial for improving patient outcomes.

## 1. Introduction

People living with HIV (PLWH) are highly susceptible to respiratory tract infections, with a frequency five times higher in individuals with a CD4 T lymphocyte count below 500 mm^3^ [1]. These infections are often polymicrobial. Viral and fungal opportunistic infections can cause pneumonia in these patients. Viruses are responsible for one-third of the cases, with rhinovirus, respiratory syncytial virus (RSV), and influenza virus being the most frequently detected. *Pneumocystis jirovecii*, a fungal agent, causes pneumonia in immunosuppressed individuals, especially those with hematological malignancies, organ transplantation, and those undergoing steroid therapy, including those with Acquired Immunodeficiency Syndrome (AIDS). PLWH make up approximately 65% of all *P. jirovecii* pneumonia (PCP) cases.

It is well known that one pathogen and its associated immune response can trigger the emergence of a second independent pathogen. In PLWH, where pneumonia is mostly polymicrobial, the rapid decrease in the number of CD4 T lymphocytes in the presence of concomitant influenza infection facilitates the emergence of PCP [3]. In light of the literature, we present a newly diagnosed AIDS patient with coinfection of influenza and PCP, diagnosed in the intensive care unit (ICU), with a focus on diagnosis, follow-up, and prognosis.

## 2. Case Report

A thirty three year old male patient presented with fever, cough, and gradually increasing shortness of breath, which began 2 weeks ago. He was hospitalized and monitored for 2 weeks at another center with a diagnosis of pneumonia but was referred to our hospital due to clinical and radiological progression. Physical examination revealed a temperature of 37°C, tachypnea, and fine crackles at the end of inspiration in the bilateral lower lung zones. His SaO_2_ was 78% and he was followed up in ICU due to hypoxemic respiratory failure.

**Figure 1:**
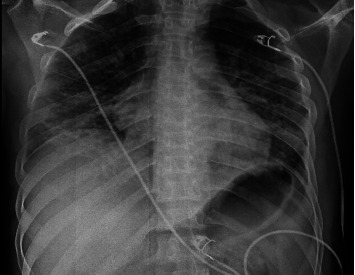
Chest radiography demonstrating bilateral interstitial infiltrations.

**Figure 2:**
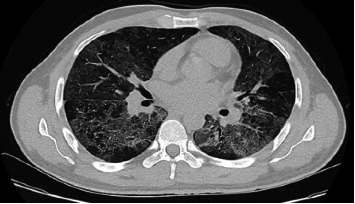
Bilateral ground glass opacities in both lungs from medial to peripheral areas on thoracic computed tomography scan.

In his medical history, it was noted that he had been diagnosed with asthma in the past 3 years. Laboratory tests showed a leukocyte count of 13,220 μL with a differential of 91.6% polymorphonuclear cells, 4.2% lymphocytes, and 3.5% monocytes; platelets of 284,000 μL; creatinine 0.6 mg/dL; aspartate transaminase 31 U/L; alanine transaminase 47 U/L; and lactate dehydrogenase 360 U/L. In the molecular respiratory panel, influenza A PCR (Biospeedy, Bioeksen, Turkey) was detected positive. Bilateral interstitial infiltrations were observed on chest radiography (Figure 1). Bilateral ground glass opacities were detected in both lungs from medial to peripheral areas on the thoracic computed tomography (CT) (Figure 2). The findings were assessed as viral pneumonia due to influenza. The patient was given oseltamivir 75 mg twice daily, ceftriaxone 1 g twice daily, and clarithromycin 500 mg twice daily.

**Figure 3:**
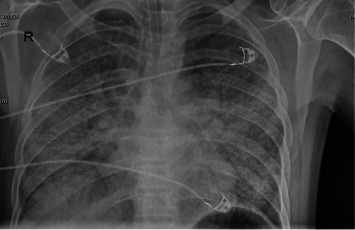
Chest *x*-ray showing radiological progression in the first week.

Further laboratory values were collected. Follow-up testing results for the anti-HIV antibody (Cobas, Roche, Germany) and the HIV1 antibody confirmation test were positive. Plasma HIV-1 RNA level was 1,150,000 cp/mL (Cobas 6800, Roche, Switzerland). CD4 T lymphocyte count was 21 μL (4.53%), and CD8 T lymphocyte count was 393 μL (87.4%). HBsAg was negative, anti-HBcIgG was negative, anti-HBs was positive, anti-HCV was negative, and anti-CMV IgG was positive. CMV DNA was 101,000 IU/mL (Cobas 6800, Roche, Germany). Sputum *P. jirovecii* DNA PCR result was positive. Sputum ARB, culture, and PCR tests performed to investigate tuberculosis were negative, and the Quantiferon assay (Quantiferon-TB Gold Plus, Qiagen, Germany) was reported as indeterminate. The patient was also was treated with trimethoprim-sulfamethoxazole (TMP-SMX) and methylprednisolone. He was intubated on the seventh day of intensive care follow-up and the fourth day of PCP treatment. Significant progression was detected in the chest radiography of the patient, who was clinically worsening (Figure 3). The findings indicated that the involvement in the middle and lower lobes has spread to include the entire lung. Ceftriaxone and clarithromycin were discontinued. Treatment regimen was escalated to meropenem and polymyxin, which covers resistant gram-negative bacteria, taking into account the surveillance data of the intensive care unit. Ganciclovir was added due to high levels of CMV DNA and bilateral interstitial pneumonia.

Ganciclovir. TMP-SMX and methylprednisolone were continued. He died due to respiratory failure within 24 h after intubation.

## 3. Discussion

Like the case we present, PLWH are more prone to severe respiratory diseases and pneumonia, which remain significant causes of morbidity and mortality. The rate of PCP is 39% in PLWH [2]. PCP begins subacutely, progressing over days to weeks, and is characterized by interstitial pneumonia with symptoms like shortness of breath, fever, and a nonproductive cough. The most crucial risk factor for PCP is the level of immunosuppression. In PLWH, risk factors for PCPinclude a CD4 T lymphocyte count of less than 200 cells/mm^3^, a history of PCP, a history of oral candidiasis, and a high plasma HIV RNA level.

Viral infections causing lymphopenia also contribute to the development of PCP. Influenza is particularly significant due to its progressive course in immunosuppressed individuals and is more severe in those with advanced-stage HIV infection [5]. It has been reported that the incidence of influenza-related hospitalization, long-term hospitalization, and the risk of in-hospital death are higher in PLWH [6]. Observed decreases in influenza-related hospitalizations with the advent of effective antiretroviral therapies (ART) suggest that the severity of seasonal influenza may be lower among patients with higher CD4 cell counts [5]. High rates of hospitalization (52%), mechanical ventilation (26%), and death (22%) were reported among 27 PLWH with confirmed H1N1 virus infection in Mexico City. 48% of patients have HIV-related pulmonary opportunistic infection, and since those who need mechanical ventilation and those who die are predominantly in this group, it is suggested that the severity of the disease may be related to the accompanying pulmonary opportunistic infection rather than influenza [7]. Based on the literatüre, it is thought that our case had a mortal course due to the development of the opportunistic infection PCP accompanying influenza infection. The absence of distinct clinical or laboratory markers differentiating PCP from influenza pneumonia complicates determining which condition is contributing to the clinical deterioration. Ultimately, the interplay between influenza and PCP makes it difficult to isolate the primary driver of respiratory failure in such cases. Clinicians must consider the entire clinical picture, including patient history, symptomatology, and results from diagnostic tests, while remaining aware of the potential for co-infection.

CD4 T lymphocytes, alveolar macrophages, and neutrophils interact in clearing *P. jirovecii* infection [8]. In influenza cases, a rapid decrease in CD4 T lymphocytes may lead to the development of PCP. Among 426 patients with confirmed pandemic H1N1 infection in China, lymphopenia was detected in 68% of adults. Leukopenia (21%) developed 2 days after the onset of the disease and returned to normal 7 days later. These transient changes in the number of peripheral blood leukocytes are related to Fas—Fas ligand signaling that induces apoptosis. Another important factor in the development of secondary infections is the inhibition of macrophage activation and phagocytosis by influenza [9].

In vitro studies have also reported that influenza A neuraminidase may increase HIV-1 replication, syncytium formation, and, as a result, CD4 T lymphocyte apoptosis and exhaustion [10–12]. In our case, the diagnosis of HIV was made during intensive care follow-up. The patient was not receiving ART, and a high viral load and CD4 T lymphocyte count of < 200 cells/mm^3^ were identified as risk factors. A report from France described a 48 year old female patient with viral suppression who had been receiving effective ART for 18 years. She had a CD4 T lymphocyte count of 644 mm^3^ and was hospitalized with a diagnosis of influenza. She was discharged after completing oseltamivir treatment. When she returned one week later with complaints of fever and shortness of breath, her SaO2 was 86%, partial oxygen pressure was 63 mmHg in arterial blood gas, lymphocyte count was 900 μL, CD4 T lymphocyte count was 216 mm^3^, and LDH level was 571 UI/L. PCP PCR was detected positive in the bronchoalveolar lavage sample. The patient was discharged after 21 days of TMP-SMX and prednisolone treatment. Similarly, a case from Italy involved an active smoker who was followed up due to asthma and HIV infection, was under viral suppression, and was hospitalized with a diagnosis of influenza A with a CD4 T lymphocyte count of 876 cells/mm^3^. On the third day of follow-up, hypoxia, acidosis, high LDH (503 UI/L), an increase in beta glucan level (> 523 pg/mL), and a decrease in CD4 T lymphocyte count (133 mm^3^) were observed. Bilateral multinodular and interstitial involvement was seen on thorax CT. TMP-SMX was started empirically. Bronchoalveolar lavage sampling yielded positive results for PCP PCR and immune fluorescence microscopy. The patient, whose treatment was completed over 3 weeks, was discharged [13]. It is known that influenza infection is more severe in PLWH, like the case we present. It is thought that the two similar cases in the literature did not progress as severely as the case we presented because HIV infection was controlled with ART in those cases. The lack of an HIV diagnosis, which hindered the incorporation of ART in our case, considerably deteriorated the prognosis.

In conclusion, community-acquired pneumonia is more common in PLWH, especially among those not receiving ART. Pulmonary infections are AIDS-defining conditions and should be screened for using an anti-HIV test. Early HIV diagnosis is important for initiation of ART and prophylaxis of opportunistic infections. In PLWH, bacterial, viral, and fungal pathogens are common causes of pneumonia, and co-infections are frequent. PCP should be considered in the presence of post-influenza hypoxic pneumonia in PLWH. Considering possible etiological agents and initiating early treatment are critical in empirical treatment approaches, significantly impacting patient prognosis.

## Data Availability

The data are not publicly available due to privacy or ethical restrictions.
